# A‐to‐I RNA editing of Filamin A regulates cellular adhesion, migration and mechanical properties

**DOI:** 10.1111/febs.16391

**Published:** 2022-03-09

**Authors:** Mamta Jain, Andreas Weber, Kathrin Maly, Greeshma Manjaly, Joanna Deek, Olena Tsvyetkova, Maja Stulić, José L. Toca‐Herrera, Michael F. Jantsch

**Affiliations:** ^1^ Division of Cell Biology Center for Anatomy and Cell Biology Medical University of Vienna Austria; ^2^ Department of Nanobiotechnology Institute for Biophysics University of Natural Resources and Life Sciences Vienna (BOKU) Austria; ^3^ 9184 Department of Physics, Cellular Biophysics E27 Technical University of Munich Garching Germany

**Keywords:** atomic force microscopy, cell adhesion, filamin A, integrin signalling, RNA editing

## Abstract

A‐to‐I RNA editing by ADARs is an abundant epitranscriptomic RNA‐modification in metazoa. In mammals, *Flna* pre‐mRNA harbours a single conserved A‐to‐I RNA editing site that introduces a Q‐to‐R amino acid change in Ig repeat 22 of the encoded protein. Previously, we showed that FLNA editing regulates smooth muscle contraction in the cardiovascular system and affects cardiac health. The present study investigates how ADAR2‐mediated A‐to‐I RNA editing of *Flna* affects actin crosslinking, cell mechanics, cellular adhesion and cell migration. Cellular assays and AFM measurements demonstrate that the edited version of FLNA increases cellular stiffness and adhesion but impairs cell migration in both, mouse fibroblasts and human tumour cells. *In vitro*, edited FLNA leads to increased actin crosslinking, forming actin gels of higher stress resistance. Our study shows that *Flna* RNA editing is a novel regulator of cytoskeletal organisation, affecting the mechanical property and mechanotransduction of cells.

## Graphical abstract

Herein, we investigated the impact of A‐to‐I RNA editing of filamin A on important cellular and mechanical processes. *Flna* pre‐mRNA harbours a single conserved A‐to‐I RNA editing site, leading to a Q‐to‐R amino acid exchange of the encoded protein. This study shows differential effects of FLNA hypo‐ (FLNA^Q^) and hyper‐editing (FLNA^R^) on cell stiffness, cell adhesion, cell migration and integrin internalization.

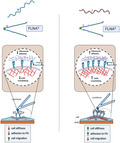

**Abbreviations**
A: adenosine
ADAR: adenosine deaminases acting on RNA
AFM: atomic force microscopy
ECM: extracellular matrix
FLN: filamin
I: inosine
Ig: immunoglobulin
M2 cells: human melanoma cells
mAb: monoclonal antibody
Q: glutamine
R: arginine

## Introduction

A‐to‐I RNA editing is an abundant, post‐transcriptional RNA‐modification event in metazoa that leads to the conversion of an **A**denosine to **I**nosine by deamination [1]. A‐to‐I editing in exons can change codons since inosines are mainly read as guanosine by the translational machinery [2]. Hence, A‐to‐I conversion in mRNAs can result in the formation of proteins that differ from the genomically encoded versions [1, 3, 4].

A‐to‐I deamination is catalysed by adenosine deaminases acting on RNA (ADARs). In mammals, ADAR1 and ADAR2 are enzymatically active [5]. ADAR1 is abundantly expressed in most tissues and primarily edits repeats in introns, 3′UTRs and non‐coding RNAs [6]. ADAR2 is only expressed in a few tissues like the nervous system, the gastrointestinal system and the vasculature where it frequently affects protein‐coding regions. Consistently, ADAR2 has been mainly shown to regulate neuronal function and more recently, the cardiovascular system [7, 8]. A‐to‐I RNA editing is a tightly regulated process and its dysregulation is known to be involved in various pathologies like neurological disorders, cardiovascular diseases and the development of cancers [9].

*Filamin A* (*Flna*) pre‐mRNA has a single, highly conserved, editing site in exon 42 leading to a Q (glutamine) to R (arginine) amino acid exchange that is conserved from birds to humans [10]. The resulting amino acid exchange is located in immunoglobulin (Ig) repeat 22 which itself is embedded in a region that serves as a scaffold for several (> 100) FLNA interacting proteins [10, 11, 12, 13]. Hence, it is likely that edited FLNA will have a different interactome than unedited FLNA. *Flna* RNA editing increases during development and is highest in blood vessels and the gastrointestinal tract of adult mice [14]. FLNA editing has also been shown to be significantly reduced in many tumour samples like bladder carcinoma and colon adenocarcinoma as compared to their normal counterparts [15].

Filamins are a family of cytoskeletal actin crosslinking proteins comprising of Filamin A (FLNA), Filamin B (FLNB) and Filamin C (FLNC) that crosslink actin filaments into subcortical, orthogonal networks [16, 17]. Filamins contain an N‐terminal actin‐binding domain followed by 24 Ig repeats, the 24th domain being the dimerisation domain. FLNA and FLNB can homo‐ and heterodimerise [18, 19]. Composite actin networks are formed as a result of crosslinking by FLNA that show significantly increased elasticity [18, 20]. FLNA mutations in humans cause neuronal and intestinal disorders [21, 22, 23]. FLNA deficient mice are embryonic lethal, showing severe cardiovascular defects and irregular vascular patterning [24, 25].

FLNA‐deficient melanoma cells show severe defects in cell migration. However, embryonic fibroblasts derived from FLNA knockout embryos do not show any effect on cell motility possibly due to compensation by FLNB function [17]. The molecular mechanism by which FLNA regulates cell migration seems multilayered since it is highly dependent on FLNA expression and its interacting partner pool in the corresponding cell line [26]. FLNA also acts as a platform for multiple protein interactions including cell membrane receptors and signalling molecules. Hence, FLNA regulates diverse cellular functions apart from regulating actin cytoskeletal organisation [27].

FLNA can act as a mechanosensor that converts extracellular mechanical signals into intracellular biochemical signals [28]. This is mediated by the ability of FLNA to bind the cytoplasmic tails of membrane proteins like integrins [13]. The integrin‐binding site of FLNA has been mapped to Ig repeat 21 that is exposed in response to mechanical force [29]. FLNA binds directly to integrin β1 with variable strength, depending on the matrix stiffness the cells are cultured on [30]. Moreover, the mechanical deformations in actin‐FLNA networks have been shown to enhance FLNA binding to integrin β7 [11]. FLNA deficiency results in integrin activation, hence FLNA expression has been shown to negatively regulate integrin activation [17]. Vimentin phosphorylation is impaired after knocking down *Flna* that directly affects integrin β1 trafficking at the cell membrane [31]. The impact of FLNA on integrin signalling or on focal adhesion turnover via vimentin‐mediated integrin recycling also plays a crucial role in both cell adhesion and cell migration [32, 33].

Atomic force microscopy studies have shown that a single molecule of FLNA can reversibly unfold and undergo stretching in response to a mechanical force [34]. Individual Ig domains within rod 2 (Ig repeats 16–24) of FLNA interact in a pairwise manner allowing domains 16 & 17, 18 & 19 and 20 & 21 to pair with each other [28]. The packing of domains 20 & 21 masks the integrin‐binding site while application of external force can open the two domains and expose the integrin‐binding site [35]. Similarly, also other FLNA‐interacting partners are implicated to be regulated by mechanical forces. In fact, the C‐terminal repeats of rod 2 are very sensitive to low forces (~ 10 pN) whereas the N‐terminal repeats in rod 1 can resist significantly higher forces [35]. Interestingly, also the F‐actin network has been shown to change its stiffness depending on FLNA concentration under the influence of large mechanical deformations [36].

Previously we showed that *Flna* RNA editing reaches 90% in human cardiovascular tissues therefore leading to prominent translation of FLNA proteins carrying a Q‐to‐R substitution at the affected amino acid 2333. Transgenic mice with impaired *Flna* editing therefore only expressing unedited FLNA^Q^ show increased smooth muscle contraction, leading to elevated blood pressure and cardiac remodelling. These data highlighted the importance of A‐to‐I RNA editing in the cardiovascular system [7]. The present work studies the impact of *Flna* A‐to‐I RNA editing on actin crosslinking, cell adhesion, migration and mechanical properties using atomic force microscopy and cell biological assays.

## Results

### Edited FLNA protein crosslinks actin more efficiently than unedited FLNA

**Fig. 1:**
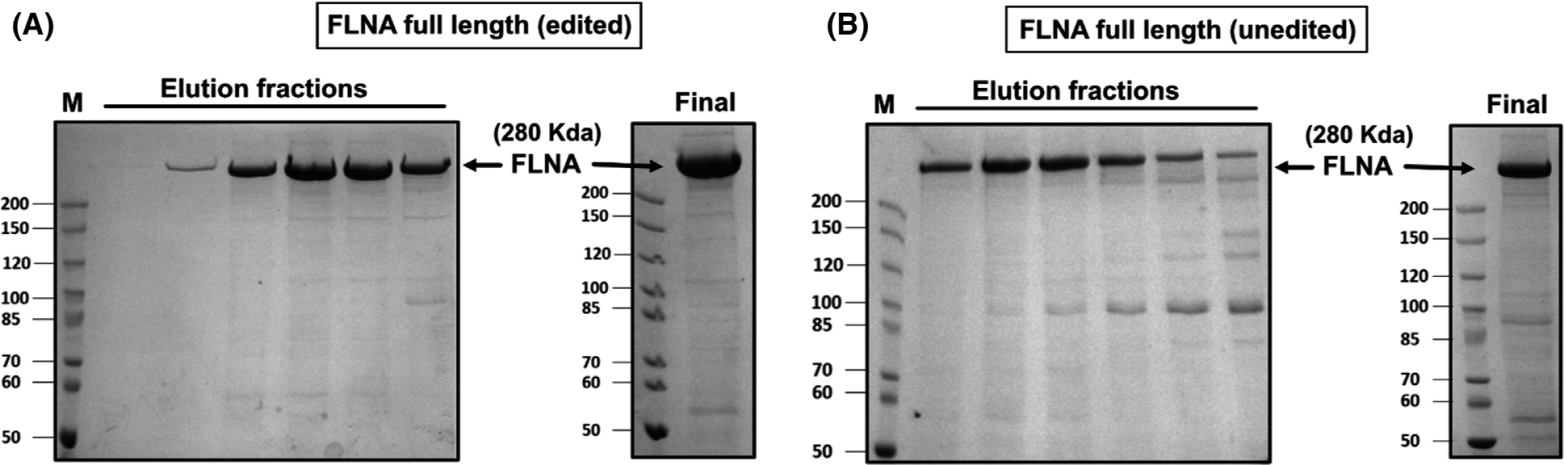
(A) Coomassie stained SDS/PAGE gel showing purified FLNA full‐length protein (edited version) purified from Sf9 insect cell system. The lanes show the pooled imidazole eluted fractions after Ni‐affinity purification. First lane shows the marker (M) lane. The final concentrated purified edited FLNA protein is shown on the right gel. (B) Coomassie stained SDS/PAGE gel showing purified FLNA full‐length protein (unedited version) purified from Sf9 insect cell system. The lanes show the pooled imidazole eluted fractions after His‐tag purification. The last lane shows the marker (M) lane. The final concentrated purified unedited FLNA protein is shown on the right gel.

**Fig. 2:**
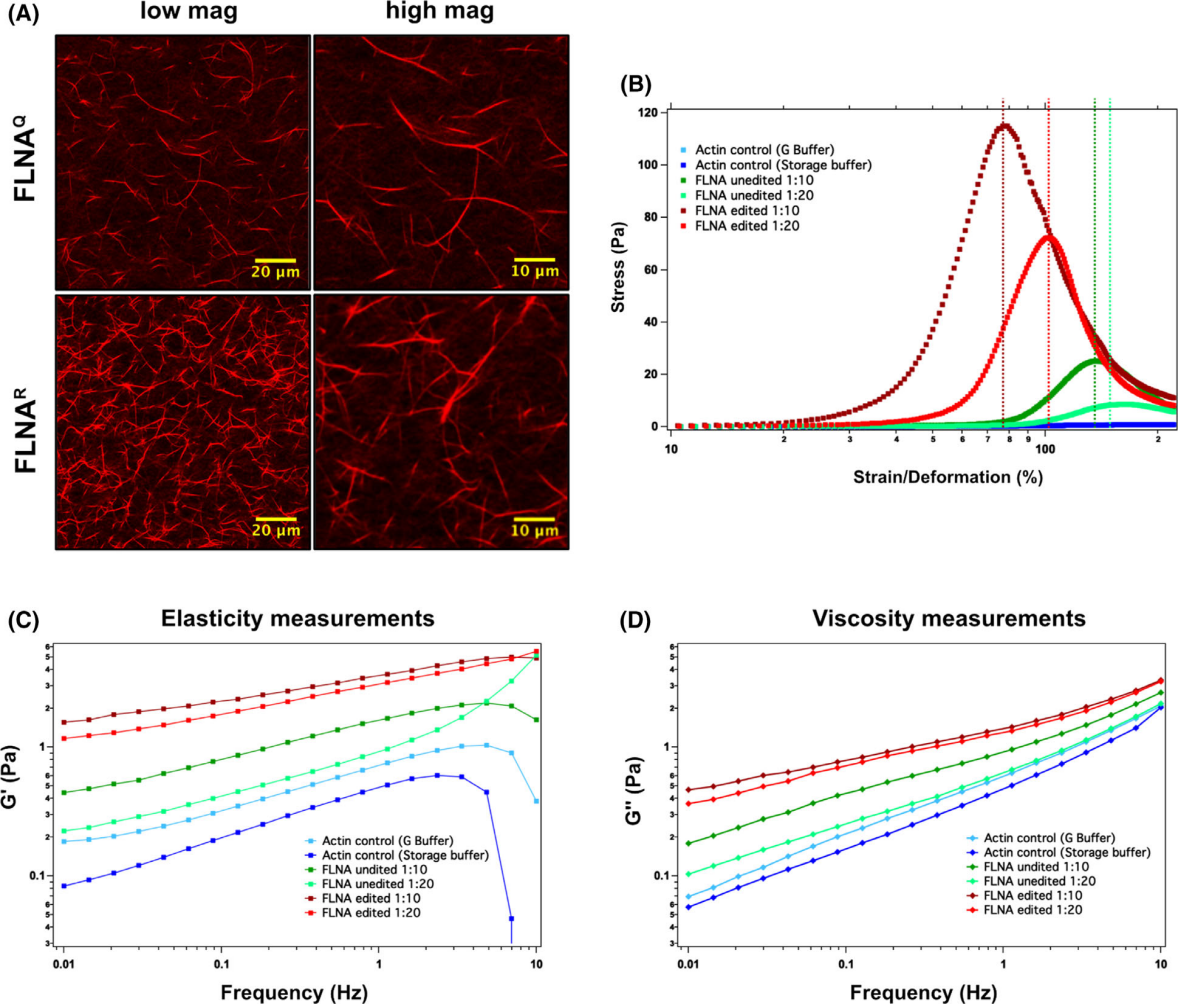
(A) Representative confocal images showing composite networks of bundled actin with unedited FLNA^Q^ (top) and edited FLNA^R^ (bottom). The low magnification (left) and high magnification (right) images are shown to allow better comparison. Scale bars indicate magnification. (B) Graph showing the non‐linear rheological behaviour demonstrating the actin bundling property of actin networks when mixed with either edited FLNA (light and dark red) and non‐edited version of FLNA (light and dark green) in 1 : 10 and 1 : 20 (FLNA : actin) ratios, respectively. Actin alone (shades of blue) in G buffer and storage buffer were used as controls. The deformation percentage is inversely proportional to the toughness of actin networks. (C, D) Graph showing the linear rheological behaviour demonstrating the elastic modulus G′ (C) and viscous modulus G″ (D) of either edited FLNA (light and dark red) and non‐edited version of FLNA (light and dark green) when mixed with actin in 1 : 10 and 1 : 20 (FLNA : actin) ratios respectively. Actin alone (shades of blue) in G buffer and storage buffer were used as controls.

FLNA is a *bona fide* actin crosslinking protein, associated with reorganisation of the cytoskeleton that also acts as a scaffold for numerous protein interactions thereby regulating various cellular functions like cell migration, adhesion and cell signalling [12, 13, 16, 17]. RNA editing by ADAR2 at a highly conserved editing site changes a glutamine codon in the pre‐mRNA to an arginine codon thereby inducing a Q‐to‐R exchange at amino acid position 2333 that is located in Ig repeat 22 of the mature protein. To test if the induced amino acid exchange can modulate the actin crosslinking properties of FLNA, we purified full‐length recombinant FLNA protein harbouring either a Q or an R at position 2333 (Fig. 1). The resulting proteins FLNA^Q^ and FLNA^R^ were mixed with F‐actin in a 1 : 10 ratio and tested for their actin bundling properties. Microscopic evaluation of actin bundles demonstrated that edited FLNA^R^ promotes the formation of denser actin networks than unedited FLNA^Q^ (Fig. 2A). Further, FLNA‐actin composite networks were subjected to rheological studies to investigate if the differences in actin crosslinking affect their deformation tolerance. The non‐linear rheological behaviour demonstrated that the edited FLNA^R^ promotes the formation of more rigid actin networks than unedited FLNA^Q^ in both 1 : 10 and 1 : 20 FLNA : actin ratios (Fig. 2B). The actin networks formed in cooperation with edited FLNA^R^ ruptured at much lower deformation than unedited FLNA^Q^‐actin networks (Fig. 2B). The linear rheological behaviour also pointed towards the higher crosslinking tendency of edited FLNA^R^ than unedited FLNA^Q^ as indicated by increased elastic modulus (G′) and as well as viscous modulus (G″) in the edited FLNA^R^ protein (Fig. 2C,D). Both, confocal microscopy‐based studies (Fig. 1A) and the rheological studies (Fig. 2B–D) show similar results confirming that FLNA‐editing promotes actin crosslinking.

### FLNA editing positively regulates cellular stiffness

**Fig. 3:**
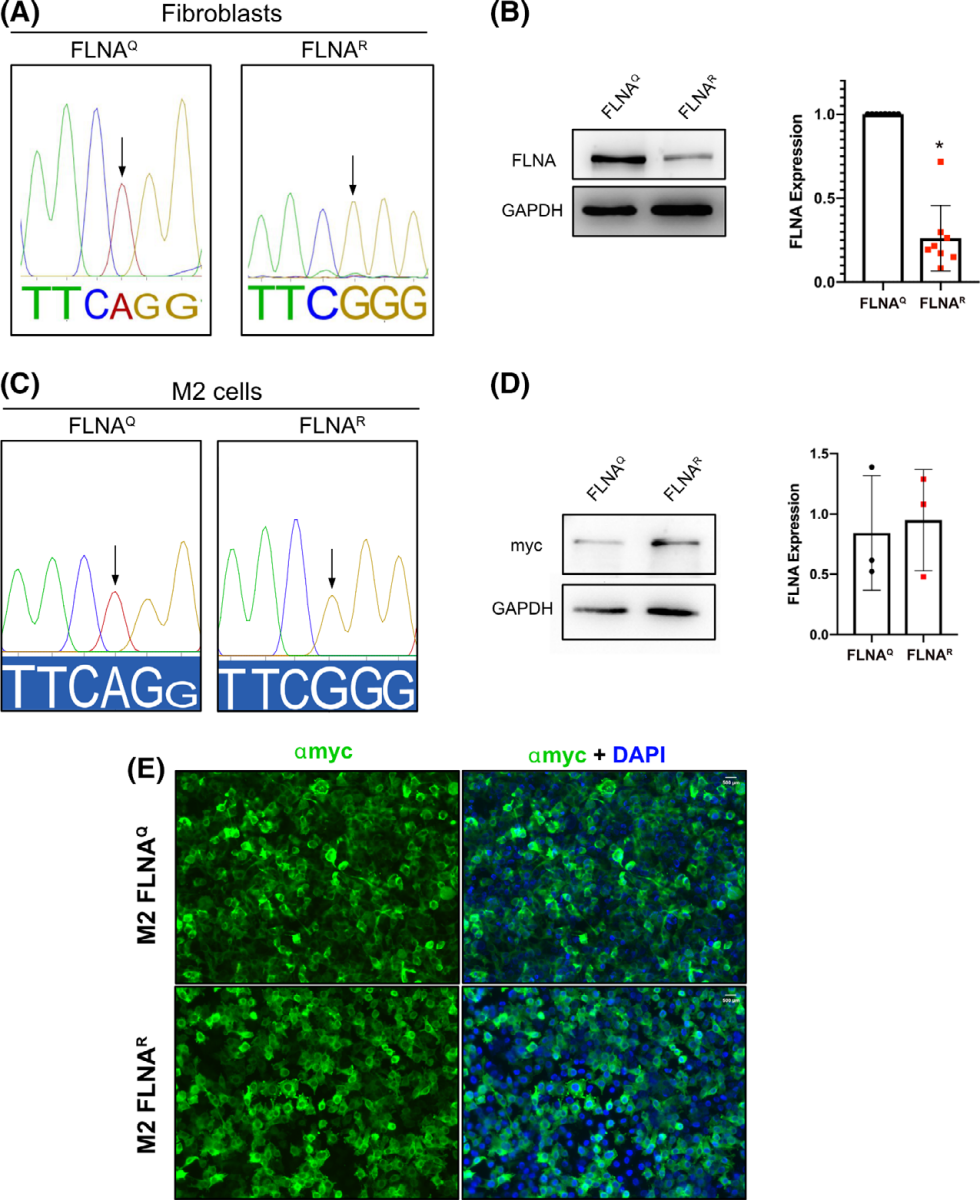
(A) Electropherograms showing FLNA editing levels in cells expressing unedited FLNA^Q^ and edited FLNA^R^. The site of the adenosine affected by editing is marked by a black arrow. (B) Western blot showing FLNA expression in FLNA^Q^ and FLNA^R^ fibroblasts. GAPDH was used as a loading control. Graph shows the quantification of FLNA expression expressed as fold‐change difference. Data shown are mean ± SD from eight independent experiments. *P < 0.05. (C) Electropherograms showing the expression of unedited and edited FLNA in melanoma (M2) cells. The adenosine normally affected by editing is marked by a black arrow. (D) Western blot showing expression in unedited FLNA^Q^ and edited FLNA^R^ in M2 cells. GAPDH was used as a loading control. Graph shows the quantification of FLNA^Q^ and FLNA^R^ expression in M2 cells. Data shown are mean ± SD from three independent experiments. The differences are non‐significant. (E) Representative images showing the expression of unedited FLNA^Q^ and edited FLNA^R^ in M2 cells by immunostaining with myc antibody (green). Nuclei are marked by DAPI (blue). Merged images in both unedited and edited samples show that a significant population of the cells express myc‐tagged FLNA protein.

Filamins can link the cytoskeleton to the extracellular matrix thus relaying extracellular mechanical signals to the cell interior, allowing mechanosensing [17, 28, 37]. Given that FLNA^R^ promotes the formation of more rigid actin‐polymers *in vitro*, we checked if FLNA editing can regulate the mechanical properties of primary mouse fibroblasts. We derived primary fibroblasts from lungs of mice exclusively expressing unedited FLNA^Q^ or pre‐edited FLNA^R^. The FLNA editing status was verified by Sanger sequencing of RT‐PCR amplicons (Fig. 3A). We also checked the fibroblasts for their protein expression and found that fibroblasts expressing FLNA^R^ show lower levels of FLNA^R^ protein than cells expressing FLNA^Q^ (Fig. 3B). As a second test system, we generated stably transfected human melanoma (M2) cells to express either unedited or edited version of FLNA. The stable M2 clones were also screened for expression of FLNA^Q^ and FLNA^R^ protein by western blotting (Fig. 3D) and immunostaining using anti‐myc mAb 9E10 (Fig. 3E). The M2 clones showed a homogeneous expression of FLNA^Q^ and FLNA^R^. The selected stable clones were checked again for FLNA editing patterns (Fig. 3C).

**Fig. 4:**
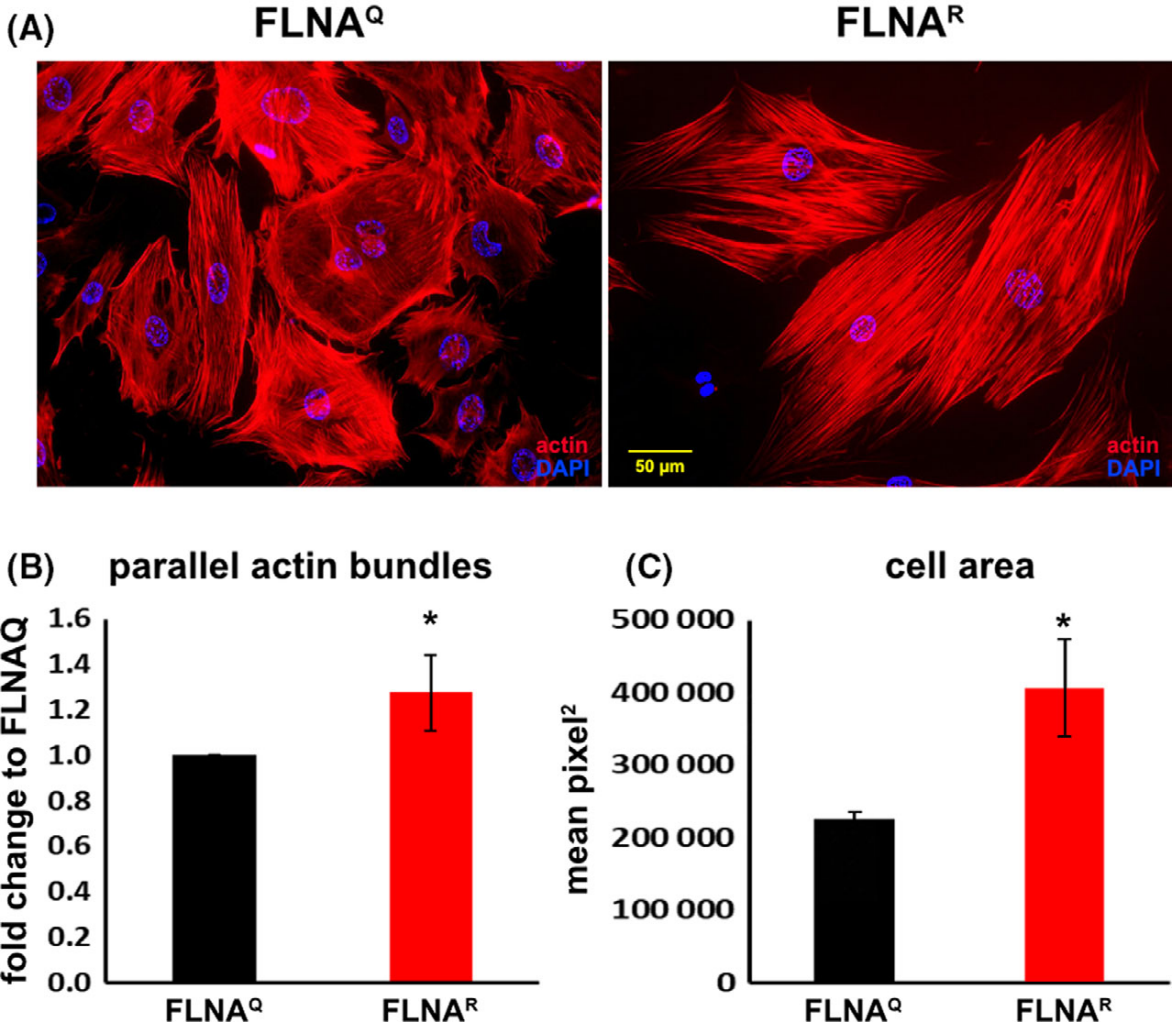
(A) Micrographs showing lung fibroblasts isolated from FLNA^Q^ (left) and FLNA^R^ mice (right), stained with phalloidin for F‐actin. A larger number of parallel actin bundles is observed in FLNA^R^ fibroblasts (B). Similarly, FLNA^R^ fibroblasts are more spread out taking a larger area (C). For raw data see Data S1 and S2. * indicates statistically significant difference of p< 0.05.

To test for *in cellulo* alterations of the actin cytoskeleton upon *Flna* editing, primary mouse fibroblasts were cultured on fibronectin for 24 h and stained with phalloidin to determine actin organisation. Interestingly, also fibroblasts expressing edited FLNA^R^, actin filaments seem more aligned when compared to fibroblasts expressing unedited FLNA^Q^ (Fig. 4A,B; Data S1). Moreover, cells expressing edited FLNA appear more spread out (Fig. 4C; Data S2).

**Fig. 5:**
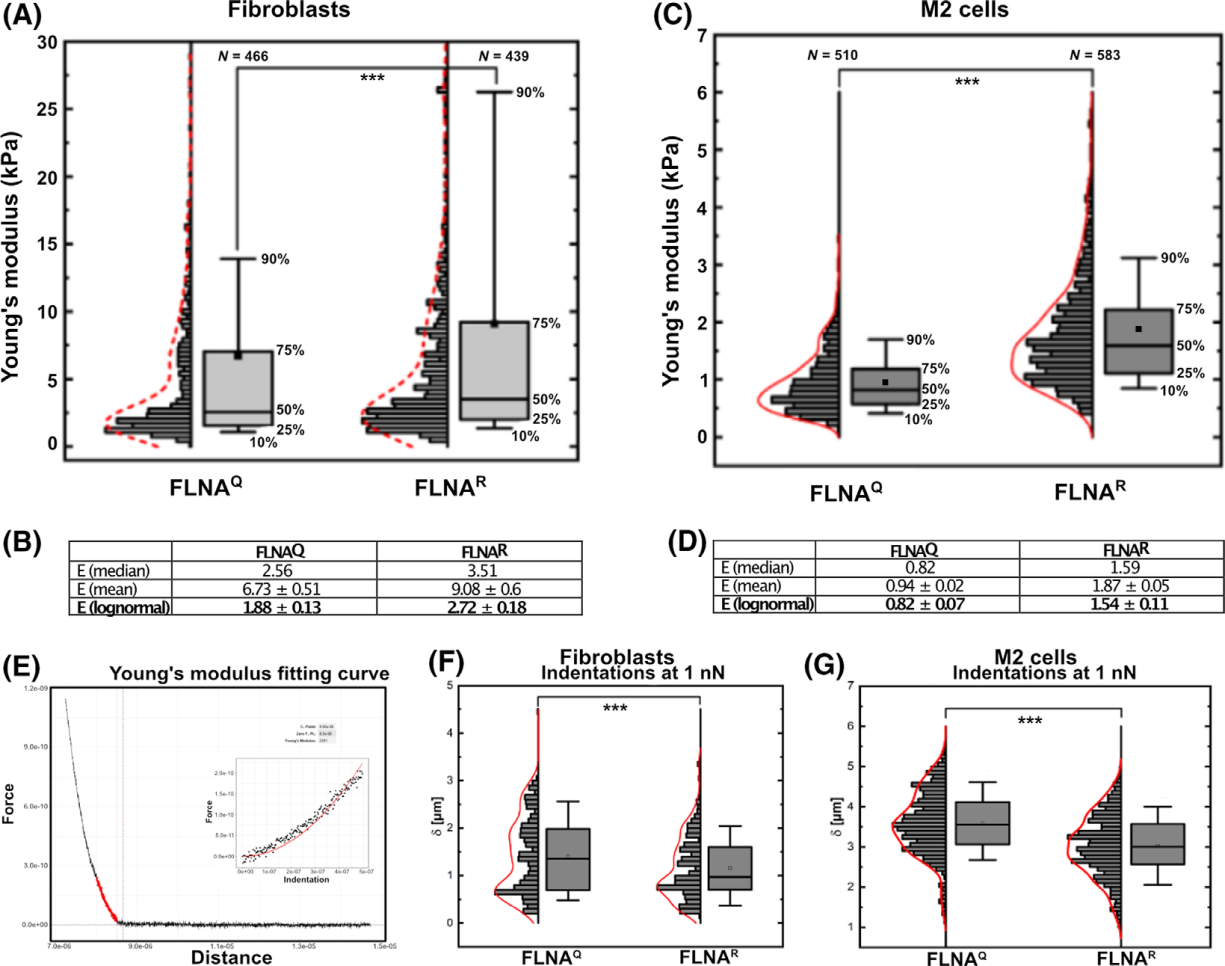
(A) Graph showing the comparison of Young’s modulus (in kPa) between FLNA^Q^ and FLNA^R^ fibroblasts at an indentation depth of 500 nm, shown as histograms and boxplots. The top and bottom whiskers correspond to the 10th and 90th percentile, the box ranges from 25th and 75th percentile. The small black square inside the boxplot represents the mean value. The fitted distribution (shown in dotted red) corresponds to a Kernel density. Number of measurements (*N*) for each sample is denoted on each boxplot. *** shows that the difference between FLNA^Q^ and FLNA^R^ is highly significant (*P* < 0.001). (B) Table showing the comparison of stiffness value between FLNA^Q^ and FLNA^R^ fibroblasts determined using different methods. The stiffness measured by any parameter (median, mean or log normal fitting) remains statistically different between the two genotypes. (C) Graph showing the comparison of Young’s modulus (in kPa) between M2 edited and unedited cells at an indentation depth of 500 nm, shown as histograms and boxplots. The top and bottom whiskers correspond to the 10th and 90th percentile, the box ranges from 25th and 75th percentile. The small black square inside the boxplot represents the mean value. The fitted distribution (red line) corresponds to a Kernel density. Number of measurements (*N*) for each sample is denoted on each box plot. *** shows that the difference between M2 edited and unedited cells is highly significant (*P* < 0.001). (D) Table showing the comparison of stiffness value between M2 edited and unedited cells determined using different methods. The stiffness measured by any parameter (median, mean or log normal fitting) remains statistically different between the two genotypes. (E) Representative indentation curve of cells showing the determination of Young’s Modulus using the R AFM toolkit. The red vertical line shows the determined contact point and the red data points show the fitted indentation region (500 nm). The inset shows the fitted function. (F) Comparison of maximum indentation δ (in μm) between fibroblasts derived from FLNA^Q^ and FLNA^R^ mice at an indentation force of 1 nN shown as histograms and boxplots. The top and bottom whiskers correspond to the 10th and 90th percentile, the box ranges from 25th and 75th percentile. The small black square inside the boxplot represents the mean value. The fitted distribution (shown in red) corresponds to a Kernel density. Number of measurements (*N*) for each sample is denoted on each box plot. *** shows that the difference between FLNA^Q^ and FLNA^R^ is highly significant (*P* < 0.001). (G) Graph comparing the maximum indentation δ (in μm) in M2 cells stably expressing edited FLNA^R^ and unedited FLNA^Q^ at an indentation force of 1 nN, shown as histograms and boxplots. The top and bottom whiskers correspond to the 10th and 90th percentile, the box ranges from 25th to 75th percentile. The small black square inside the boxplot represents the mean value. The fitted distribution (red line) corresponds to a Kernel density. Number of measurements (*N*) for each sample is denoted on each box plot. *** shows that the difference between M2 edited and unedited cells is highly significant (*P* < 0.001).

Next, fibroblasts and M2 cells were tested for their mechanical properties using atomic force microscopy (AFM). Measurements could be well fitted using elastic theory to determine the Young’s Modulus (Fig. 5E). The Young’s Modulus measurements (in kPa) were taken at an indentation of 500 nm. These experiments showed that fibroblasts expressing edited FLNA^R^ are much stiffer (45%) than their counterparts expressing unedited FLNA^Q^ (Fig. 5A), despite FLNA^R^ cells expressing less FLNA protein. For both cell lines, the Young’s Modulus values were lognormal distributed with a most probable value of 1.88 kPa for FLNA^Q^ and 2.72 kPa for FLNA^R^. The comparison of stiffness values was determined using different statistical parameters of the distributions (Fig. 5B) and were found significantly different by all of them (median, mean or log normal fitting) *P* < 0.001.

Human M2 stable clones when subjected to similar studies showed that M2 cells expressing edited FLNA^R^ are around 90% stiffer than M2 cells expressing unedited FLNA^Q^ as shown by the Young’s modulus measurements (in kPa) taken at an indentation of 500 nm using AFM (Fig. 5C,E). The stiffness values were comparable and statistically significant when determined by different parameters (Fig. 5D). In accordance with the Young’s Modulus, stiffer cells expressing FLNA^R^ show lower indentations than softer cells expressing FLNA^Q^ at a maximum load of 1 nN in both fibroblasts (Fig. 5F) and human melanoma cells (Fig. 5G). Overall, these results show that FLNA RNA editing positively regulates stiffness of mouse and human cells.

### Repeat 22 of unedited FLNA^Q^ protein unfolds under lower force than FLNA^R^ repeat 22

**Fig. 6:**
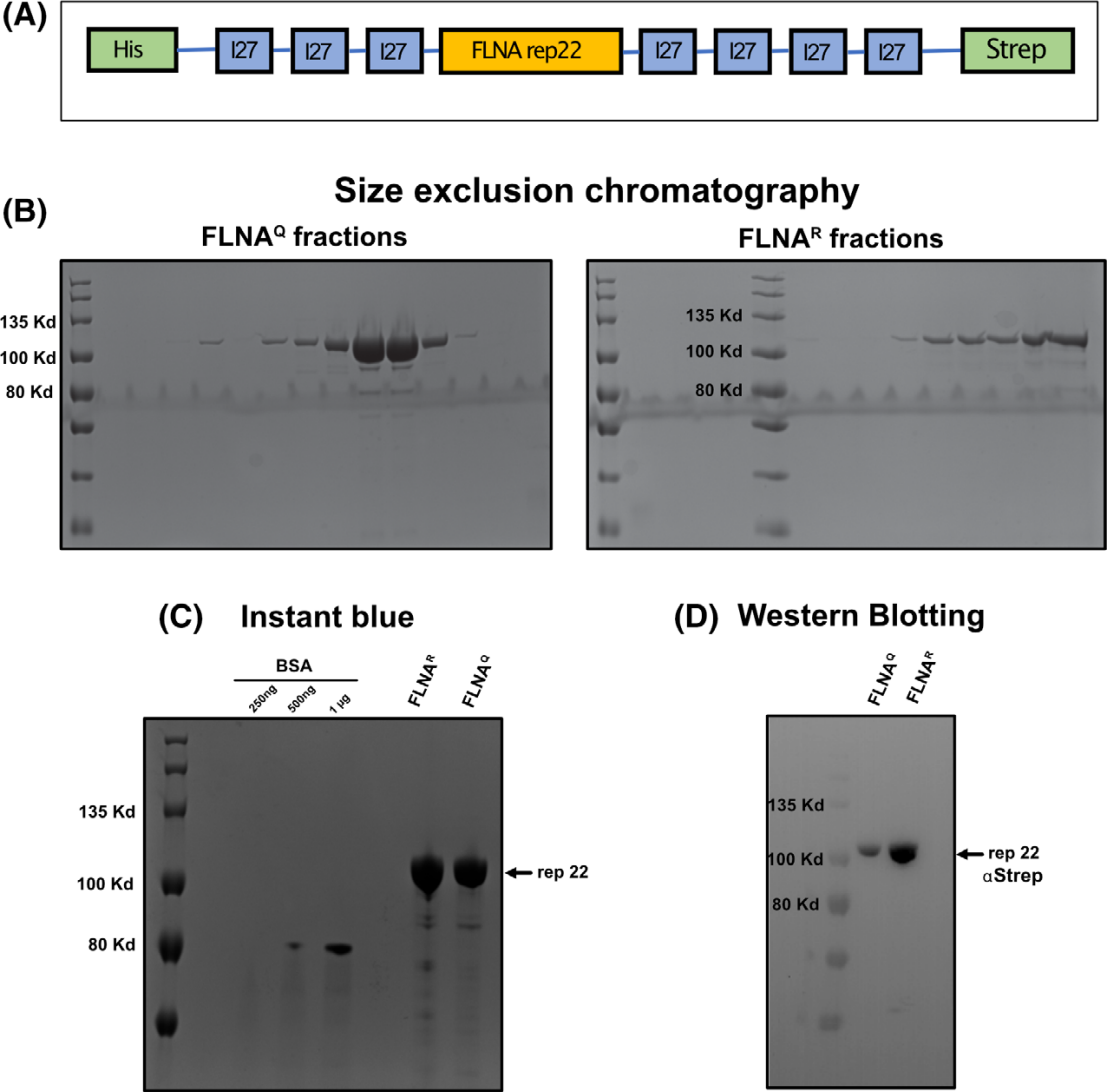
(A) Scheme showing a construct containing FLNA repeat 22 flanked by 3 I27 titin domains at the N‐terminus and 4 I27 domains at the C‐terminus. The expression construct harbours a His tag at the N terminus and a Strep tag at the C terminus. (B) SDS/PAGE protein gels stained with Coomassie showing the purified fractions obtained after affinity chromatography followed by size exclusion chromatography of recombinant proteins containing repeat 22 of FLNA^Q^ and FLNA^R^. (C) SDS/PAGE protein gels stained with Coomassie showing the purified fractions pooled and concentrated and loaded with known amounts of BSA for densitometric analysis. (D) Western blot confirming the presence of protein at the expected size for both FLNA versions probed with α‐strep antibody.

Intramolecular foldings and resulting mechanical properties of FLNA domains – especially repeats 16–23 have been shown to play an important role not only in maintaining actin‐tension but also in regulating interactions with other proteins like integrins or signalling molecules [38]. Previous studies suggest that the mechanical property of FLNA domains can affect the tensile strength and rheological behaviour of actin networks [35, 36]. We wanted to know if there are differences in the mechanical properties of Ig repeat 22 of FLNA where RNA editing leads to a single Q → R amino acid exchange. We cloned both Q and R versions of FLNA rep 22 flanked by 3 titin I27 domains at the N terminus and 4 titin I27 domains at the C terminus (Fig. 6A). The engineered protein was expressed and purified via a strep‐tag and further polished by size exclusion chromatography, concentrated and quantified (Fig. 6B,C). The protein was also verified by western blotting using an α‐strep antibody (Fig. 6D).

**Fig. 7:**
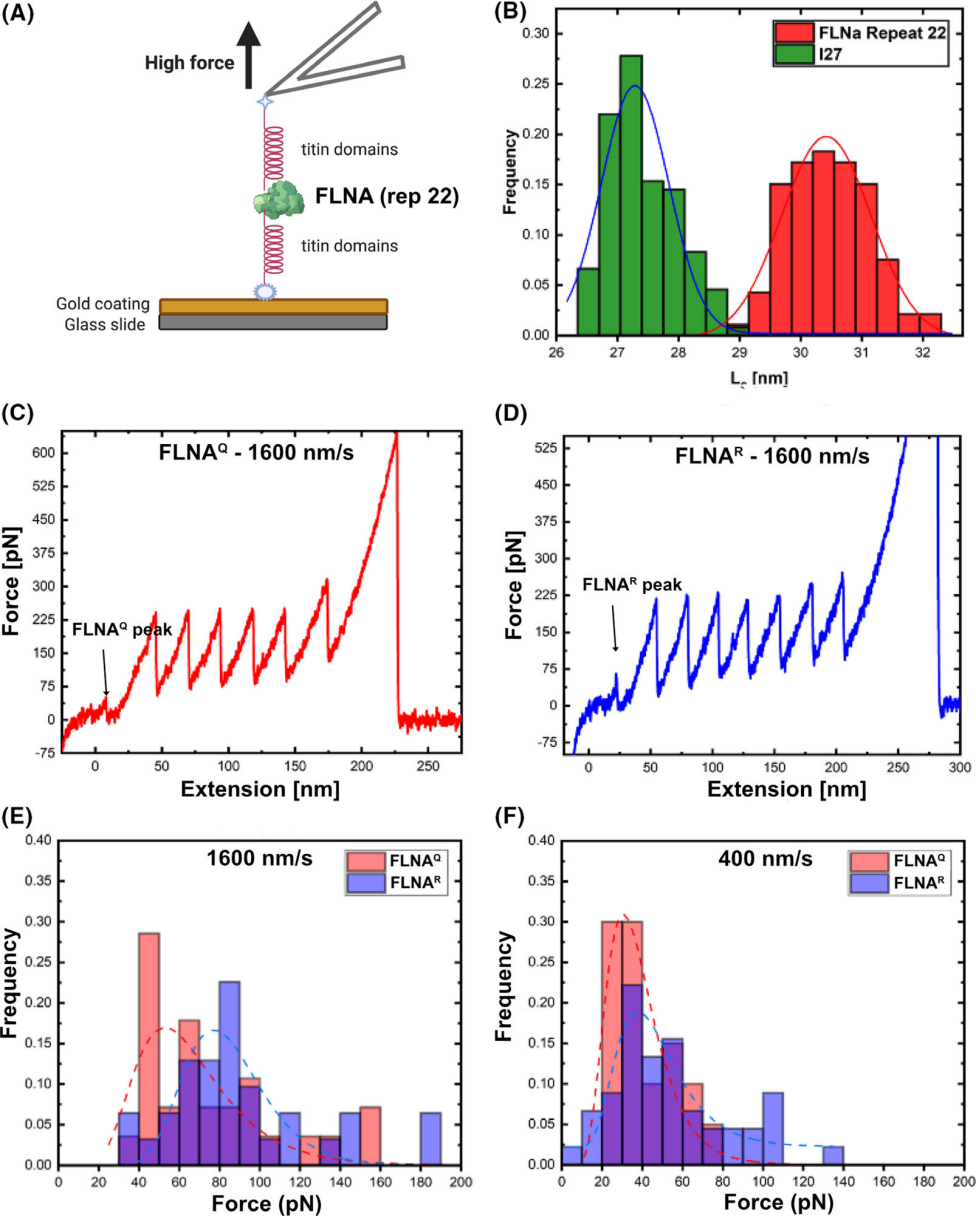
(A) Scheme showing the AFM cantilever mechanically unfolding the FLNA protein (repeat 22) encompassed by titin domains at both ends. FLNA recombinant protein is fixed to the glass slide using a gold coating. (B) Comparison of contour length of protein domains compared across I27 titin domain alone (green) and FLNA repeat 22 (red). As can be seen, a distinction between I27 titin domain (contour length of 27.3 ± 0.13 nm) and FLNA repeat 22 domains (30.4 ± 0.2 nm) is possible. The fitted curves show lognormal distributions. (C) Example measurement of the mechanical unfolding of a I27‐I27‐I27‐FLNA^Q^‐I27‐I27‐I27‐I27 polyprotein. The first peak with a higher contour length and a low unfolding force corresponds to the FLNA repeat domain. The repetitive peaks after the FLNA peak show the unfolding of the individual titin I27 domains. (D) Example measurement of the mechanical unfolding of a I27‐I27‐I27‐FLNA^R^‐I27‐I27‐I27‐I27 polyprotein. (E) Probability distribution of FLNA unfolding forces at 1600 nm·s^−1^ represented by stacked bar graph. The fitted curves show lognormal distributions. (F) Probability distribution of FLNA unfolding forces at 400 nm·s^−1^ represented by stacked bar graph.

Using AFM‐mediated elasticity measurements we were able to discriminate FLNA peaks in the single molecule unfolding tracks according to the contour length of approximately 30.4 ± 0.2 nm from I27 peaks showing a contour length of 27.3 ± 0.1 nm (Fig. 7A,B). Due to the low force needed for unfolding FLNA repeat 22, such peaks appeared as first peaks in the unfolding plots (Fig. 7C,D respectively). AFM experiments performed with the purified recombinant proteins showed that FLNA^Q^ unfolds at a significant lower force of 61.4 ± 7.4 pN whereas FLNA^R^ unfolds at 82.0 ± 7.0 pN measured at 1600 nm·s^−1^ (Fig. 7E). Additional experiments performed at a lower unfolding rate of 400 nm·s^−1^ showed lower unfolding forces of 35.4 ± 4.1 pN for FLNA^Q^ and 43.7 ± 5.1 pN for FLNA^R^ – as predicted by Bell‐Evans theory [39, 40] (Fig. 7F). The obtained values agree with already published unfolding forces for FLNA fragments when normalised for the unloading rate, showing that FLNA repeat 22 is a protein that unfolds under relatively low forces in the range of tens of pN [34, 35, 38, 41]. Interestingly, the two rod segments of Filamin A show differential mechanical stability, with rod 2 harbouring repeats 16–23 being most susceptible to unfold at low forces of ~ 10 pN [35]. Further, different groups have shown independently that domain‐domain interactions between Filamin A repeats in rod 2 seem to impose a structural hierarchy of the unfolding forces, an effect that could play an important role in the mechanical function of Filamin A [35, 41, 42, 43].

### FLNA editing reduces cell migration in mouse fibroblasts and human melanoma cells

**Fig. 8:**
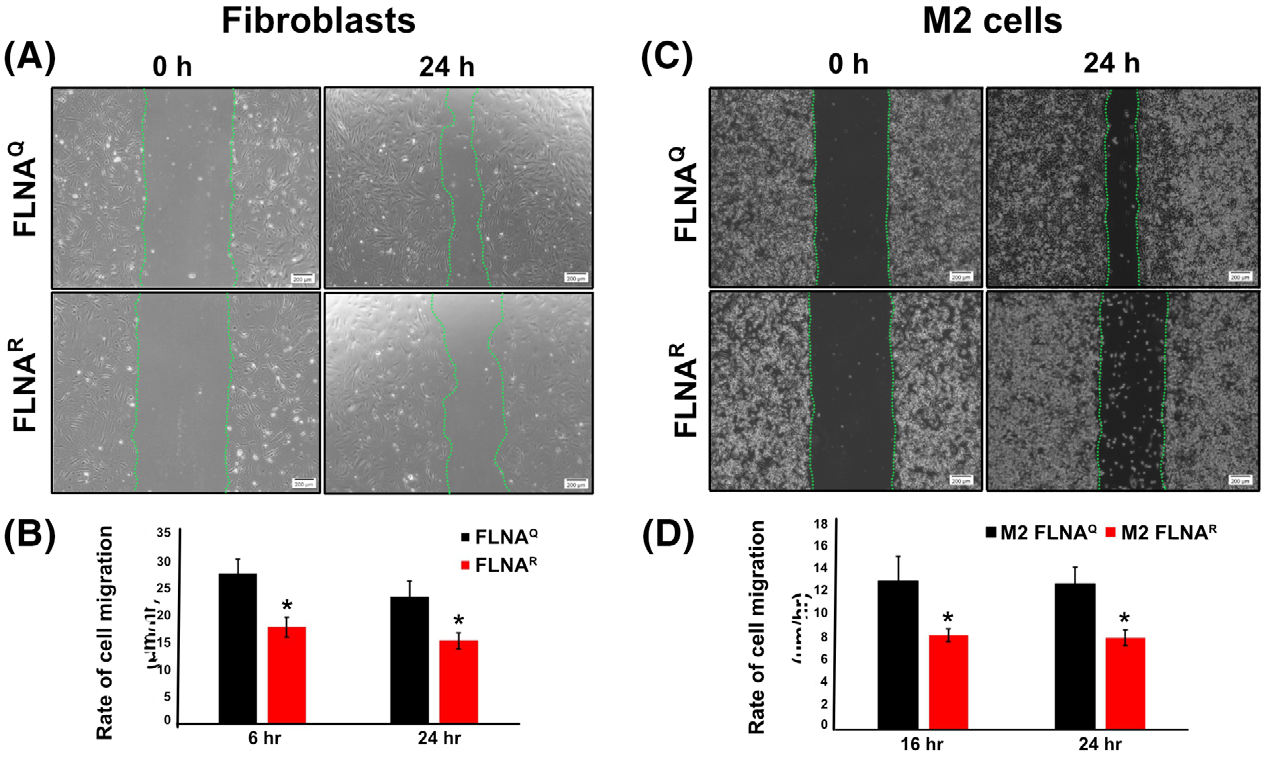
(A) Representative images showing wound‐healing based cell migration assays using FLNA^Q^ and FLNA^R^ fibroblasts. The images are taken at 0 and 24 h time points. The green dotted line represents the wound margin of the scratched area. Scale bar = 200 µm. (B) Bar graph showing the quantification of rate of cell migration between FLNA^Q^ and FLNA^R^ fibroblasts at 6 and 24 h time points. Data shown as mean ± SD from three independent experiments. *P < 0.05. (C) Representative images showing wound‐healing based cell migration assay using M2 cells expressing FLNA^Q^ or FLNA^R^. The images are taken at 0 and 24 h time points. The green dotted line represents the wound margin of the scratched area. (D) Bar graph showing the quantification of rate of cell migration between FLNA^Q^ and FLNA^R^ M2 cells at 16 and 24 h time points. Data shown as mean ± SD from three independent experiments. *P < 0.05.

Several studies show that FLNA regulates cell migration and tumour invasion by controlling focal adhesion turnover [26, 33, 44, 45, 46, 47]. Although the role of FLNA in regulating cell migration is well studied, it is still elusive to which extent editing of *Flna* RNA affects cell motility. Therefore, the role of FLNA editing in cell motility, was investigated by cell‐scratch migration assays in both mouse lung fibroblasts and human tumour (M2) cells, expressing either FLNA^Q^ or FLNA^R^, plated on fibronectin. These assays clearly demonstrated that mouse fibroblasts expressing FLNA^R^ migrate at much lower velocity and are consequently less competent to close a wound scratch than fibroblasts expressing FLNA^Q^ within a 24 h time period (Fig. 8A,B). Retarded migration in fibroblasts expressing FLNA^R^ could already be seen at 6 h (Fig. 8B). Also, a cell scratch assay performed on human M2 cells expressing FLNA^Q^ or FLNA^R^ confirmed that cells expressing FLNA^R^ migrate much slower than M2 cells expressing FLNA^Q^ (Fig. 8C,D). Due to the slower cell migration seen in M2 cells, measurements were taken at 16 and 24 h after scratch induction (Fig. 8D).

**Fig. 9:**
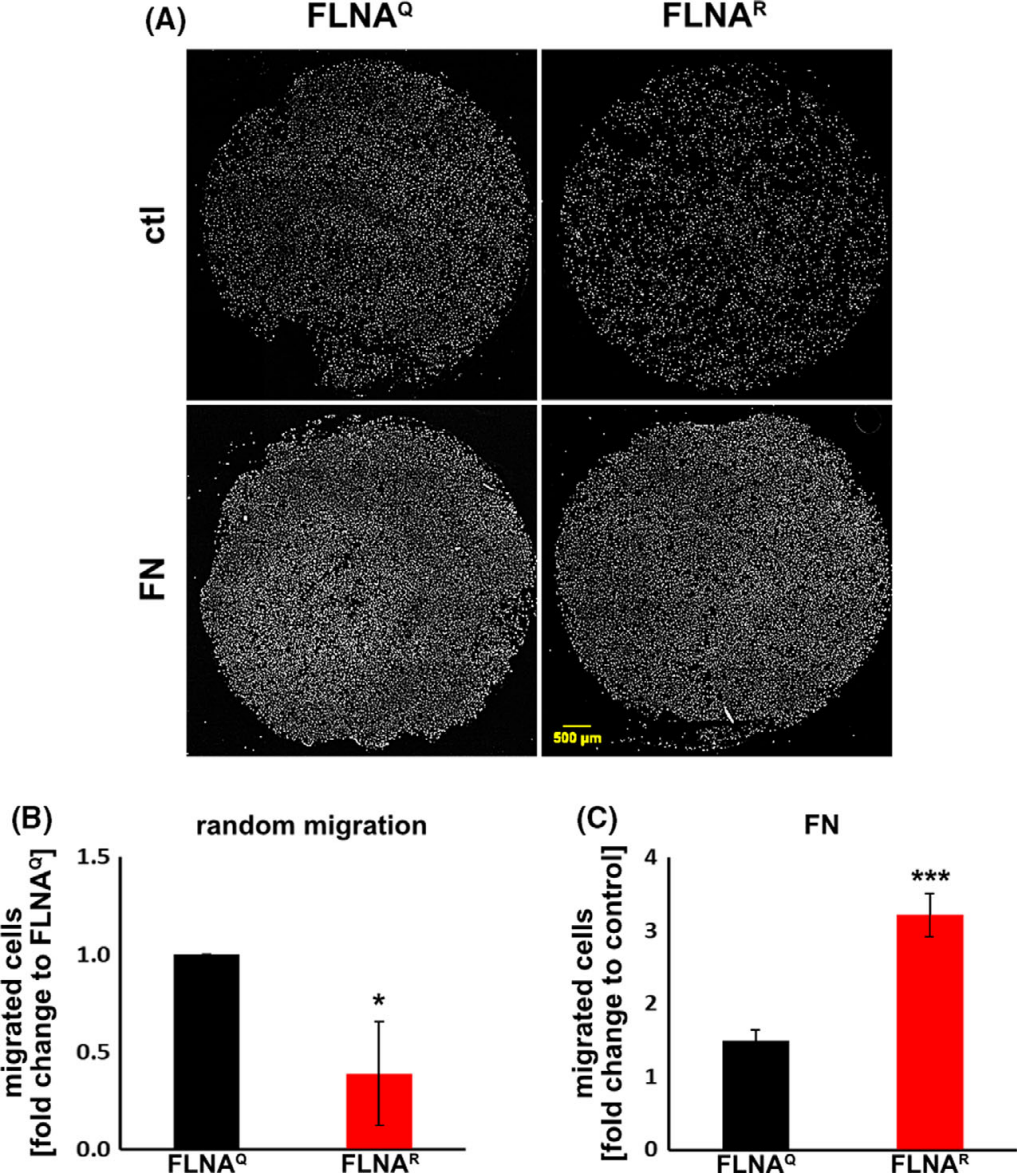
Transwell assays reveal altered cell migration and chemoattraction depending on the FLNA editing status. Primary mouse fibroblasts were seeded into transwell plates. After a defined incubation period the top of the transwell unit was cleaned and the migrated cells at the bottom of the membrane were stained and counted. (A) In the presence of standard medium FLNAQ cells show a faster migration rate (top and B). When fibronectin was added to the lower chamber as a chemoattractant FLNAQ cells showed a significantly higher migration (bottom and C). (B, C) Quantification of migrating cells.

Additionally, a chemotaxis assay was performed, using the transwell‐system to investigate the random migration capacity in more detail. To do so, an equal number of primary mouse fibroblasts isolated from lungs of FLNA^Q^ or FLNA^R^ mice was seeded onto gelatine‐coated transwell‐inserts in the presence of standard medium. Again, fibroblasts expressing FLNA^Q^ were more motile compared to fibroblasts expressing FLNA^R^ (Fig. 9A,B). Next, cells were attracted with fibronectin for 24 h. Interestingly, fibronectin‐induced migration was higher in fibroblasts expressing FLNA^R^ than in those expressing FLNA^Q^ (Fig. 9A,C). These results show that RNA editing of FLNA controls the rate of cell migration in mouse fibroblasts and human tumour cells. The reduced migration rate of fibroblasts expressing FLNA^R^ on fibronectin coated dishes as well as the increased response to soluble fibronectin raised the question if an altered integrin‐binding might be responsible for these phenomena.

### Fibroblasts expressing FLNA^R^ show increased adhesion to fibronectin

**Fig. 10:**
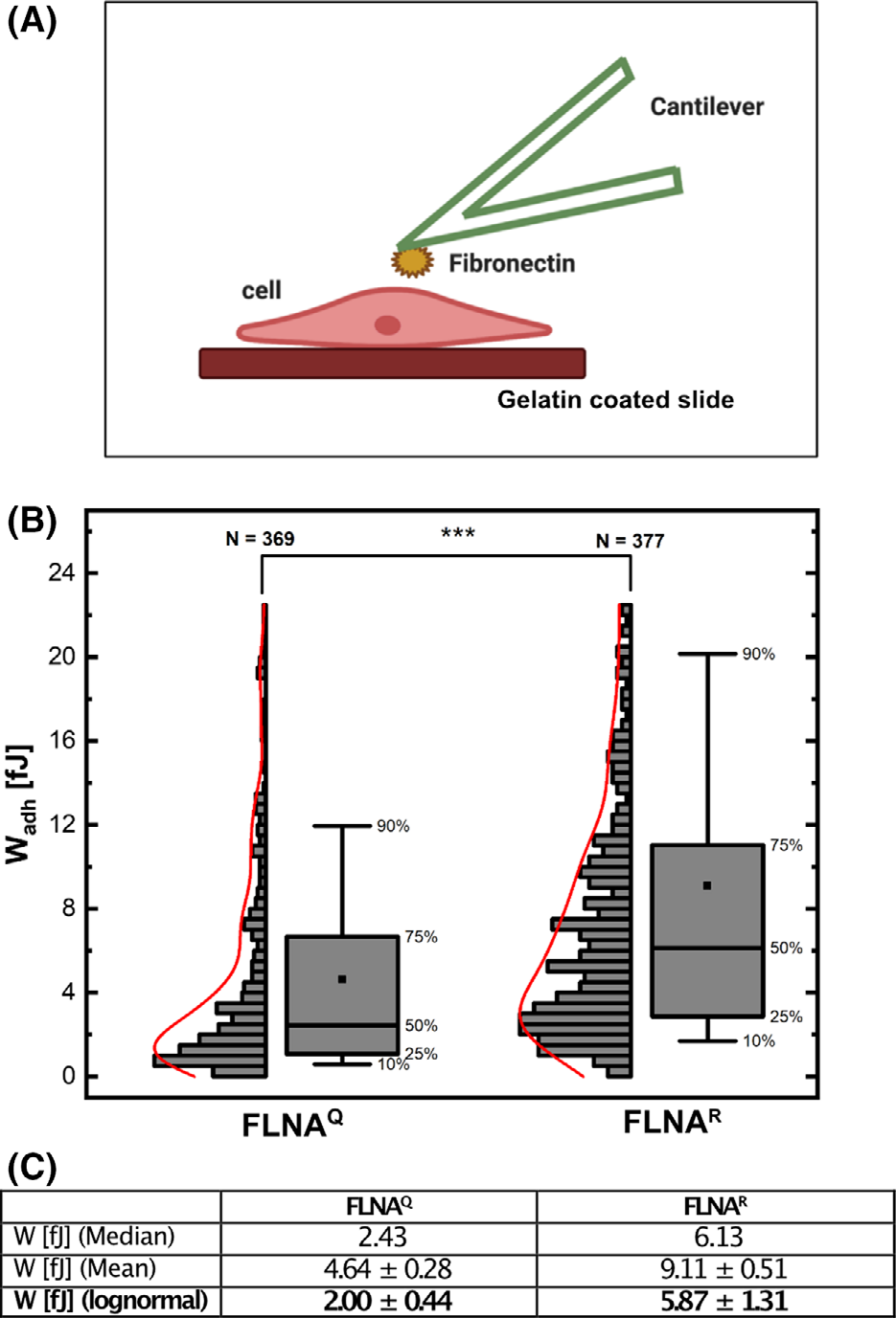
(A) Scheme showing force measurements using a fibronectin coated cantilever on single cells. The cells are grown on 0.1% gelatine‐coated microslides used for AFM measurements. (B) Graph showing the comparison of work of adhesion (in fJ) between FLNA^Q^ and FLNA^R^ fibroblasts measured as interaction with fibronectin coated cantilever, shown as histograms and boxplots. The top and bottom whiskers correspond to the 10th and 90th percentile, the box ranges from 25th and 75th percentile. The small black square inside the boxplot represents the mean value. The fitted distribution (shown in red) corresponds to a Kernel density. Number of measurements (*N*) for each sample is denoted on the top of each box plot. ***P < 0.001 by non‐parametric Mann–Whitney test. (C) Table showing the comparison of work of adhesion between FLNA^Q^ and FLNA^R^ fibroblasts determined using different methods. The adhesion force measured by any parameter (median, mean or log normal fitting) remains statistically different between the two genotypes.

Fibronectin is an important extracellular matrix (ECM) component that plays a critical role in cell adhesion and motility [48, 49]. To test whether FLNA RNA editing is also affecting cell adhesion, we used tipless, fibronectin‐coated cantilevers to compare the strength of adhesion of FLNA^Q^ and FLNA^R^ fibroblasts using AFM. As expected, the work required to break the adhesion between a fibronectin coated cantilever and the respective cell type showed that FLNA^R^ fibroblasts interact ~ 2.5 times stronger with fibronectin than fibroblasts expressing FLNA^Q^ (Fig. 10). These results show that increased FLNA editing strengthens cellular adhesion to the extracellular matrix protein fibronectin.

### Fibroblasts expressing edited FLNA^R^ show elevated integrin levels

**Fig. 11:**
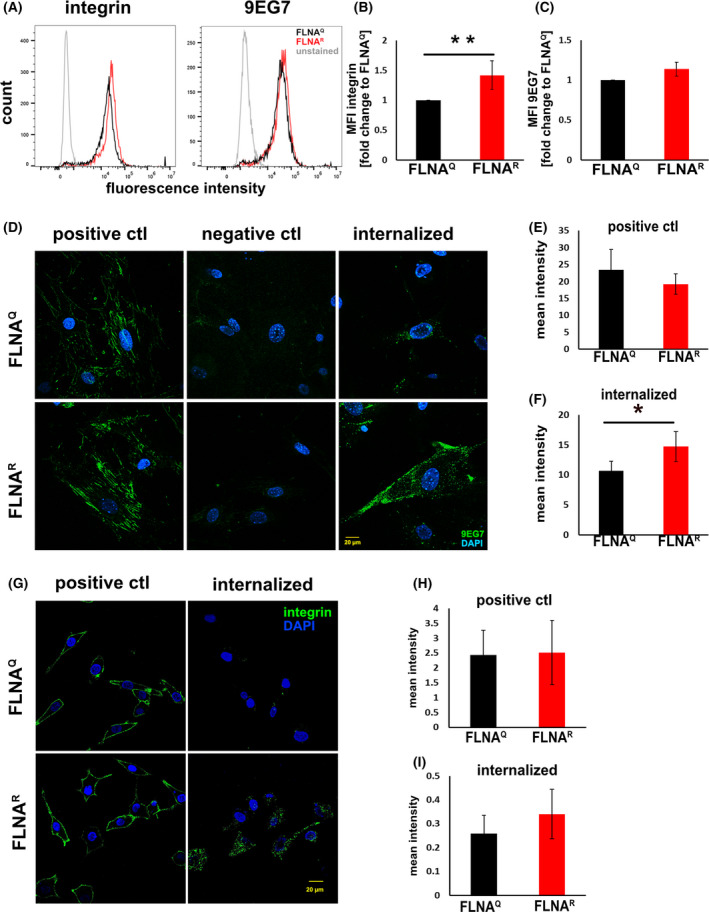
(A) Representative FACS histograms of FLNA^Q^ and FLNA^R^ lung fibroblast stained for total, surface expressed integrins (left) or activated integrin (right), indicating increased expression of total integrin on FLNA^R^ fibroblasts. (B) Quantitation of three biological replicates of the experiment shown in A, indicating increased expression of integrin in FLNA^R^ cells (**P < 0.05). (C) Biological triplicates indicate that levels of activated integrin are only slightly increased in FLNA^R^ cells. (D) Representative images showing integrin (mAb 9EG7) staining in the positive control (cells incubated with extracellular domain specific antibody at 4 °C only), negative control (incubated with antibody at 4 °C followed by an acid wash) and after 60 min internalisation at 37 °C. The FLNA^Q^ fibroblasts are shown on top and fibroblasts expressing FLNA^R^ are shown at the bottom. Nuclei are stained with DAPI. Scale bar = 20 μm. (E) Graph showing the quantification of integrin expression in the positive control, comparing FLNA^Q^ and FLNA^R^ fibroblasts. (F) Graph showing the quantification of internalised integrin signal after 60 min compared between FLNA^Q^ and FLNA^R^ fibroblasts. Data shown as mean ± SD from three independent experiments. *P < 0.08. (G) Immunofluorescent staining of surface expressed FLNA in non‐permeabilised M2 cells stably expressing FLNA^Q^ or FLNA^R^ (positive control) and staining of internalised integrin after uptake of antibody on permeabilised cells (internalised) (H) Quantification of three biological replicates of quantification of surface expressed integrin, indicating equal expression of integrin in FLNA^Q^ and FLNA^R^ cells. (I) Quantification of biological triplicates indicate that internalised integrin signal after 60 min compared between FLNA^Q^ and FLNA^R^ fibroblasts. FLNAR cells show a slight trend towards increased integrin internalisation that is not significant (*P* < 0.3). Raw data can be found in data file 3. Scale bar = 20 μm.

A major receptor of fibronectin is integrin α5β1 that not only regulates matrix formation but also plays an important role in controlling cell migration [50]. Integrins are known to regulate cell adhesion and migration by linking the extracellular matrix to the cytoskeletal network [51, 52, 53]. Moreover, FLNA controls cell motility by binding to integrin receptors [54]. Since fibroblasts expressing edited FLNA^R^ showed enhanced adhesion to fibronectin and reduced cell migration, we tested if FLNA editing could regulate integrin levels and activation. Therefore, levels of total and activated integrin β1 were determined by flow cytometry and immunocytochemical staining of unedited and edited fibroblasts plated on fibronectin. mAb 9EG7 was used to specifically detect activated integrin β1 [55]. Interestingly, flow cytometry revealed that fibroblasts expressing FLNA^R^ express significantly more total integrin β1 compared to fibroblasts expressing FLNA^Q^ (Fig 11A,B). Also, the level of activated integrin was increased in FLNA^R^ cells, albeit non‐significantly (Fig 11C). To further investigate, if internalisation might influence the level of activated integrin, immunocytochemical staining was performed. As expected, fibroblasts of both genotypes show comparable signal intensities of activated integrin β1. Still, fibroblasts expressing FLNA^R^ show more internalised receptor than FLNA^Q^ cells, albeit at non‐significant levels (Fig. 11D,E,F; Data S3). The same trend was obtained when investigating M2 cells stably expressing edited and unedited FLNA, although the increase in integrin internalisation was not significant for FLNA^R^ cells (Fig. 11G,H,I). Overall, these results show that cells expressing edited FLNA^R^ also show increased surface integrin expression, slightly elevated integrin activation and a trend towards higher integrin internalisation than cells expressing unedited FLNAQ. Together, this may explain the observed differences in cellular migration and adherence to fibronectin‐coated surfaces.

## Discussion

FLNA provides mechanical rigidity to the cellular cortex while acting as a mechanosensor that links the extracellular matrix to the actin cytoskeleton. The mechanotransducing role of FLNA along with its ability to act as a scaffold for interacting with more than 100 diverse proteins allows FLNA to control many cellular functions including cell adhesion, migration and signalling [56, 57]. In this study, we show that RNA editing of FLNA affects many of these cellular features.

While the amino terminus and parts of rod 1 are required for actin binding, the C‐terminal rod 2 of FLNA is mostly interacting with membrane proteins and regulators of cellular contraction [58]. Interestingly, our results show that RNA editing of *Flna* leading to a Q‐to‐R exchange in repeat 22 of rod 2 domain protein regulates its *in vitro* actin crosslinking ability. Actin‐FLNA gel networks made with edited FLNA^R^ protein were significantly stiffer and could tolerate more strain induced deformation. The mechanism by which the single amino acid change in the 22nd repeat of the protein can affect actin crosslinking remains elusive at this point. It is possible, however, that the angle at which FLNA dimers crosslink actin could change as a result of the Q‐to‐R exchange thereby leading to the formation of more prominent bundles induced by FLNA^R^. Similarly, as we could show that FLNA^R^ is more resistant to mechanical unfolding it seems possible that actin networks formed with FLNA^Q^ show a higher deformability as indicated by the rheology experiments shown here. In any case, future studies will be required to unequivocally address this point.

Cell mechanics play a crucial role in mechanotransduction as well as cell migration, and dysregulation in mechanical properties of cells have been shown to be involved in defects in wound healing and progression of inflammation and cancer [59]. The mechanical property of cells determines how they respond to different mechanical environments and how these can propagate into functional signalling inside the cell [60, 61, 62]. Our results show that the edited version of FLNA that is capable of enhanced actin crosslinking also promotes cellular stiffness. FLNA‐deficient cells have been shown to be softer and display reduced contractile forces [63]. In our study, both mouse fibroblasts and human tumour cells show a significant increase in cellular stiffness when the edited version of FLNA, FLNA^R^ is expressed.

Mouse‐derived fibroblasts express less FLNA^R^ than FLNA^Q^. Still, FLNA^R^ fibroblasts display increased stiffness when compared to FLNA^Q^ fibroblasts. Consistently, in M2 cells where comparable amounts of edited FLNA^R^ and unedited FLNA^Q^ are expressed, an even stronger increase in stiffness of FLNA^R^ expressing cells can be observed. These results demonstrate that cellular stiffness is indeed increased by the editing‐induced Q‐to‐R exchange in FLNA. Increased cellular stiffness is also consistent with the more organised actin bundles observed in FLNA^R^ cells.

**Fig. 12:**
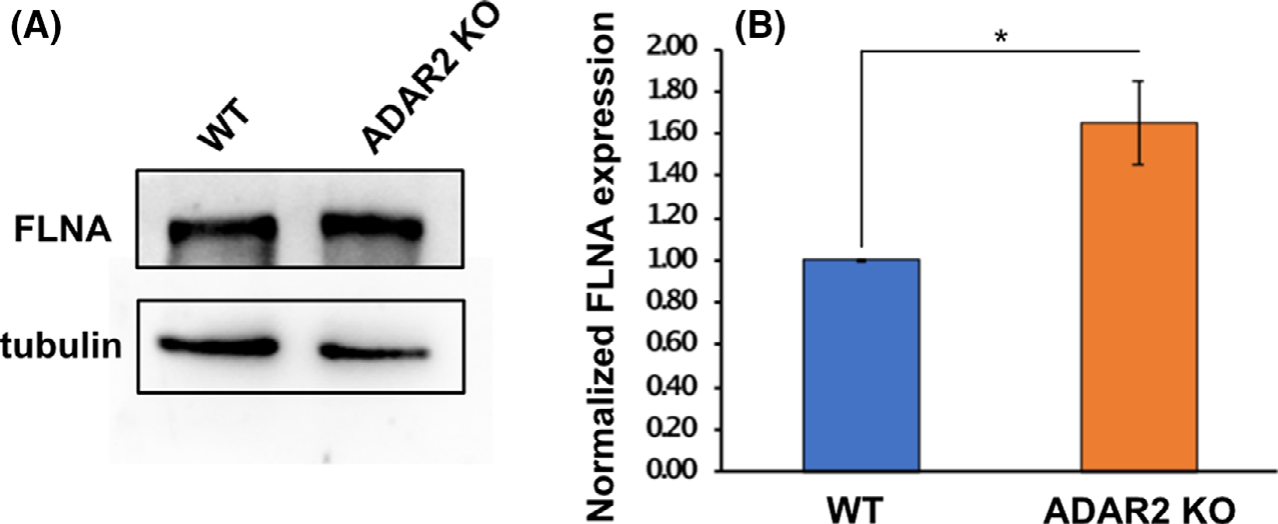
(A) Western blot showing FLNA expression in WT and ADAR2 KO fibroblasts using polyclonal FLNA antibody. Tubulin was used as a loading control. (B) Graph shows the quantification of normalised FLNA expression expressed as fold change difference between WT and ADAR2 KO fibroblasts. Data shown are mean ± SD from three independent experiments. *P < 0.05.

Since mouse cells in which only constitutively edited FLNA^R^ is expressed from the endogenous locus produce less FLNA^R^ than mouse cells only expressing FLNA^Q^ we were wondering whether editing itself can affect FLNA levels in mice. We therefore compared the expression levels of FLNA in tissues where FLNA editing is high, in both wild‐type and editing‐deficient ADAR2 knockout mice [64]. Interestingly, ADAR2 deficiency leads to increased FLNA expression, indicating that editing leads to a decrease in FLNA expression, at least in some tissues (Fig. 12). Most likely, the decrease in expression is the effect of reduced FLNA‐splicing observed upon pre‐mRNA editing [65]. In any case, it is remarkable that FLNA^R^ leads to increased cellular stiffness, despite its seemingly reduced expression in mouse fibroblasts.

Mechanical unfolding of paired domains in the rod 2 region of FLNA has been shown to influence the binding partner preference. It was suggested that mechanical stability of interdomains of repeat 16–23 might also be important for regulating actin network stiffness and tensile force generation [35]. Our finding that edited FLNA^R^ repeat 22 is unfolding at significantly higher force than the unedited repeat 22 might contribute to the greater stiffness and crosslinking degree of FLNA^R^‐actin gels. Unedited FLNA^Q^ repeat 22 might lead to a greater flexibility of this domain or change the angle at which FLNA can crosslink actin fibres. This, in turn, might regulate the rheological and mechanical properties of the F‐actin cytoskeletal networks investigated here. Clearly, further studies will be required to test if the differential mechanical unfolding of repeat 22 will also control the pool/strength of binding partners like integrins. Moreover, structural studies will highlight differences of rod 2 segments of FLNA^R^ or FLNA^Q^.

Our results also show that cells expressing FLNA^R^ are not only stiffer but also migrate at reduced speed. This would be consistent with FLNA^R^ cells displaying higher movement‐resisting forces [66]. It thus seems, that FLNA editing provides a link between cell mechanics and actin network tension in regulating cell migration.

Cell‐matrix interaction is an integral part of the cell migration process. Cell‐matrix adhesion sites generated by migratory cells are required to probe the surrounding microenvironment and to generate the suitable migratory forces required for movement [67]. The AFM cell adhesion experiments show that FLNA^R^ fibroblasts more strongly interact with fibronectin which can also explain their reduced migration in scratch wound‐healing assays. Integrins play a crucial role in cell migration. The extracellular domain of integrins bind to a specific sequence motif present in extracellular matrices like fibronectin and collagen that brings conformational changes in the receptor and initiates the downstream receptor signalling [68]. Integrin expression, activation and turnover due to intracellular trafficking are important features to regulate integrin adhesion complexes involved in cell adhesion and migration [67]. Our results show increased integrin expression and elevated internalisation in edited cells. The presence of more internalised integrin indicates a higher integrin turnover, suggesting that edited FLNA^R^ promotes the formation of integrin adhesion complexes. Together, our results demonstrate that FLNA editing links actin crosslinking, adhesion dynamics, integrin expression and internalisation. It will be interesting to test if FLNA editing also regulates the interaction between FLNA and integrins.

**Fig. 13:**
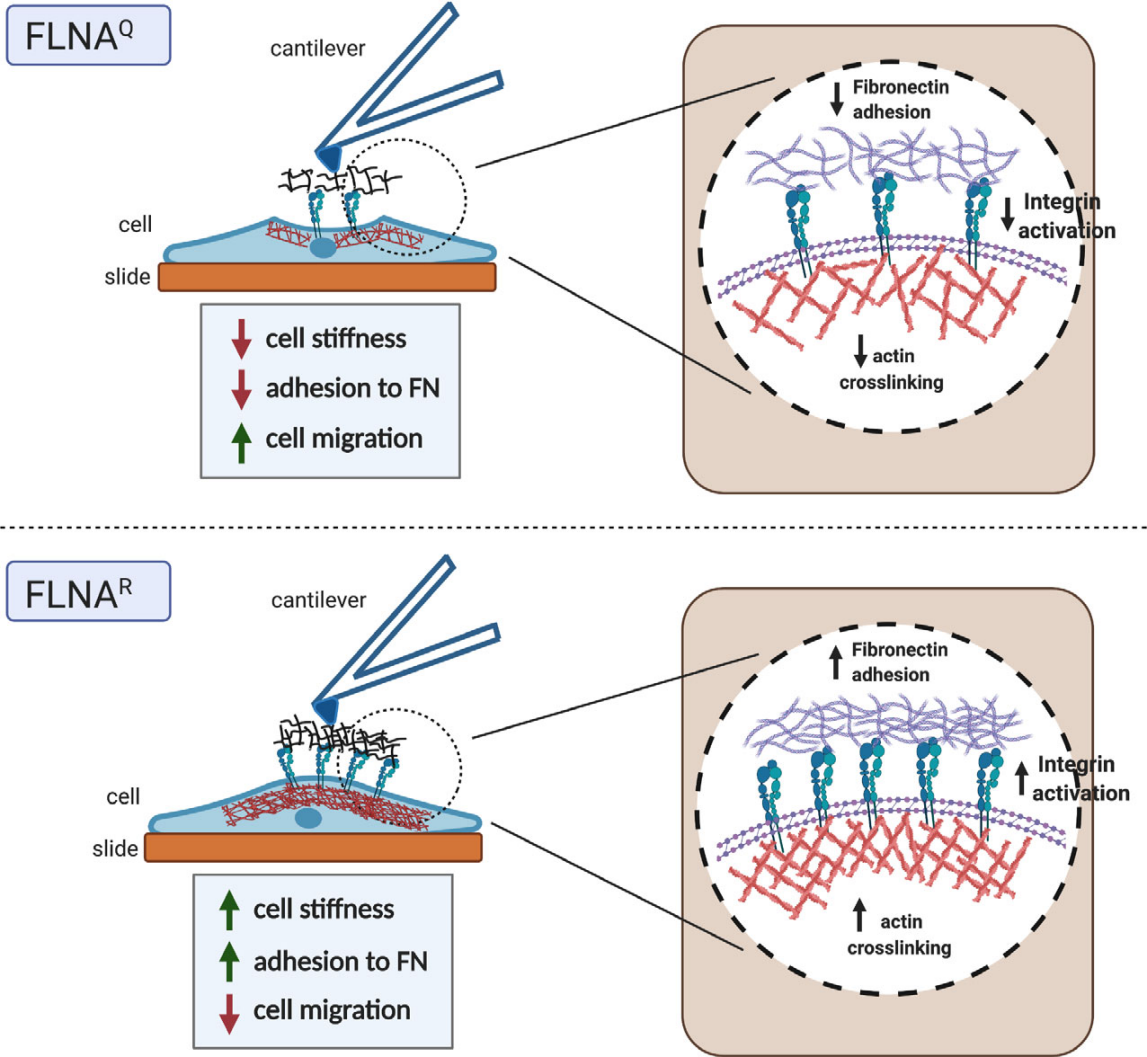
Model showing the role of FLNA editing in cell stiffness, adhesion, migration, integrin activation and actin crosslinking. (Top) Lack of FLNA editing leads to a reduction in cell stiffness, decrease in cell adhesion to fibronectin and an increase in cell migration. FLNA editing deficient cells show less integrin internalisation. The actin crosslinking to FLNA protein also remains low when FLNA is in unmodified form. (Bottom) The pre‐edited version of FLNA leads to increased cell stiffness, enhanced adhesion to fibronectin and reduced cell migration properties. Edited FLNA shows stronger actin crosslinking. Fibroblasts expressing the edited version of FLNA display much more receptor internalisation.

Overall, our study shows that RNA editing of *Flna* pre‐mRNA can act as an important integrator of extracellular mechanical signals and intracellular biochemical signals. Edited FLNA^R^ crosslinks more actin and produces tougher FLNA‐actin gels. Mouse fibroblasts and human tumour cells expressing edited FLNA are stiffer, adhere stronger to fibronectin, likely as a result of more organised actin bundles and increased integrin expression, thus resulting in reduced cell migration (Fig. 13). Our study therefore highlights a novel link between cell mechanics and mechanotransduction. Our results also strengthen previous observations showing that changes in elasticity of actin cytoskeletal networks impact both mechanical properties of cell and adhesion dynamics, with *Flna* editing being a key regulator. With the help of advanced genetic tools, induced modulation of *Flna* RNA editing also offers a promising tool to be used to regulate cell migration in processes like wound healing and tumour metastasis.

## Materials and methods

### Actin crosslinking assay

A plasmid containing the unedited human FLNA cDNA was a kind gift of T. Stoessel (Harvard medical school). The edited nucleotide was converted from an A to a G using site‐directed mutagenesis. The resulting plasmids were recloned via the gateway system into a His‐tagged baculoviral vector (Invitrogen, Carlsbad, CA, USA). His‐tagged full‐length protein was expressed in S2 cells. The recombinant full‐length proteins were purified via Ni affinity columns and eluted with an imidazole gradient. The actin crosslinking assay followed by confocal microscopy and the rheology studies were done as described previously [69, 70]. Briefly, 10 µm of G‐actin prepared from rabbit skeletal muscle was mixed with 1 µm of either edited or unedited versions of purified, recombinant full‐length FLNA protein (1 : 10 ratio) and gelsolin was added to the mixture to induce actin polymerisation. The actin filaments from the polymerised gels were stained with phalloidin (SIGMA, St. Louis, MO, USA) and subjected to confocal microscopy on a Leica TCS SP5 to visualise the actin networks (Leica, Wetzlar, Germany). Rheology measurements were done on a stress‐controlled rheometer (Physica MCR 301; Anton Paar, Graz, Austria) following the protocol described previously [69]. For linear as well as non‐linear rheology experiments, two ratios of FLNA : actin were used: (a) 1 : 10 ratio that corresponded to 1 µm of FLNA and 10 µm of actin; (b) 1 : 20 ratio that corresponded to 0.5 µm of FLNA and 10 µm of actin. All experiments were done using a final G buffer comprising of 40 mm Tris, 100 mm KCl, 50 mm NaCl, 5 mm ATP, 2 mm MgCl_2_, 2 mm CaCl_2_, 2 mm DTT, 2 mm β‐mercaptoethanol, pH 7.5. Storage buffer used as another control was the FLNA protein storage buffer comprising of 50 mm Tris‐HCl, 100 mm NaCl, 5 mm β‐mercaptoethanol pH 8.

### Lung fibroblasts isolation

Primary fibroblasts were derived from the lungs of 6 weeks old FLNA^Q^ and FLNA^R^ male mice. Briefly, lungs were isolated and washed with ice‐cold PBS. Lung tissues were minced under sterile conditions and incubated with 0.5 mg·mL^−1^ of collagenase I solution (C2674 SIGMA) on a rotating wheel at 37 °C for 1 h. After 1 h, digested tissue was further homogenised through a 18 G syringe needle and filtered through a 70‐μm cell strainer (Sarstedt, Germany). The filtrate was centrifuged at 300 ***g*** for 5 min, the supernatant was discarded and the cell pellet was resuspended in DMEM medium containing 20% FBS and plated on 0.1% gelatine‐coated dishes.

### M2 stable cell line generation

Human melanoma (M2) cells were chosen for stable cell line generation since they do not express FLNA protein [71]. To generate the stable M2 cells, plasmids harbouring myc‐tagged full‐length unedited or pre‐edited human FLNA that also carried a hygromycin resistance cassette were linearised using XbaI and transfected in M2 cells using Lipofectamine 2000 (Invitrogen). After 24 h of transfection, cells were incubated with 100 μg·mL^−1^ hygromycin‐containing medium and selected for 10 days post‐hygromycin addition. After 10 days, single clones were picked and transferred to a 96‐well plate. The clones were grown to confluence, expanded to two wells and harvested for genomic DNA isolation. Clones were then checked by PCR and Sanger sequencing to confirm the expression and editing status of FLNA. Pure clones were expanded and checked for myc expression both by western blotting and immunofluorescence staining. Clones that showed similar myc expression levels were used for both cellular and atomic force microscopy‐based experiments.

### Mechanical characterisation of cells

For Atomic Force Microscopy (AFM) measurements, 10^5^ cells were cultured on 24 mm diameter glass slides coated with 0.1% gelatine for 24 h. Before measurements, cells were washed with PBS, and then the medium was changed to Hepes‐buffered Leibovitz media. Cells were placed in a BioCell sample stage at 37 °C in media. Measurements were performed using a JPK Nanowizard III with a piezo z‐range of 15 µm equipped with an inverted optical microscope using a 10× air objective. Each sample was used for a maximum of 3 h. For indentation experiments, soft triangular cantilevers (DNP‐S, B, Bruker, Billerica, MA) with a four‐sided pyramidal tip with a nominal radius of below 20 nm, a nominal spring constant of 0.12 N·m^−1^, and a resonance frequency of 23 kHz in air (around 5 kHz in liquid) were used. Cantilevers were cleaned before measurements using UV/ozone and then calibrated applying the thermal noise method making use of the equipartition theorem [72]. Samples were let to equilibrate prior to measurements for 30 min. For indentation experiments, cells were measured with a loading rate of 5 µm·s^−1^, at a maximum force of 1 nN (maximum indentation depending on cell line), a curve length of 7.5 µm (10 µm for the M2 cell lines) and a sample rate of 4096 Hz. At least 50 cells were measured 10 times per cell with at least two independent samples. In between cells, glass was measured to ensure cantilever cleanliness. Measurement settings as well as the number of measurements were chosen according to a recent publication [73].

Outlier curves were removed after visual examination using the jpk dp software (Berlin, Germany). Curves were then contact point fitted, baseline corrected, indentation calculated, and zero slope corrected using a lab written routine in the programming language r [74, 75]. An elastic mechanical model following Hertz theory with Sneddon extension for pyramidal indenters was fitted to Force–distance curves, following

(1) $$F=\frac{E}{1‐\nu^{2}}\frac{\tan\;\alpha }{\sqrt{2}}\delta^{2}$$

with an indentation of 500 nm and a Poisson ratio set to 0.5 (cells assumed as incompressible materials). Cells are complex viscoelastic bodies that here can still be described by simpler elastic theory using the approximations of small indentations (below 10% of cell height), isotropic properties and an infinitely hard indenter. In addition, the indentation at a force of 1 nN was calculated.

### Cell migration assays

Fibroblasts or M2 cells were counted, plated on fibronectin coated dishes (10 μg·mL^−1^) and grown till confluence. The monolayers were then scratched using a 200‐μL yellow tip. The cells were washed and incubated with reduced (5%) serum medium. The cells were imaged at 0, 6 and 24 h for fibroblasts and 0, 16 and 24 h for M2 cells. M2 cells migrate much slower than fibroblasts, hence a 16 h time point was chosen to allow optimal detection of migration differences. Both cell types were imaged with an inverted tissue culture microscope (Olympus CKX53, Olympus Europe, Hamburg, Germany) attached to a 5 megapixel digital camera (Olympus SC50) using cell sens entry software. The distance between the scratch was measured using imagej [76] at each time point and the rate of cell migration (distance/time) was calculated and compared across the two genotypes at each time point in both the cell lines.

The transwell‐system with a pore size of 8 µm was used in combination with 24‐well plates to investigate the effect of fibronectin‐induced chemotaxis on primary fibroblasts. First, 600 µL DMEM containing 0.1% BSA (serum‐free) with 20 µg·mL^−1^ fibronectin were added to the lower compartment of the transwell‐unit. As a negative control, serum‐free medium was added in the lower compartment. Fibroblasts were washed twice with PBS before the migration assays. 5 × 10^4^ cells in 200 µL medium containing 0.1% BSA were transferred to the upper compartment of the inserts (coated with 0.1% gelatine). After 24 h incubation at 37 °C, the cells within and on the lower side of the PET membrane were fixed with 4% PFA at RT. Non‐migrated fibroblasts on the upper side of the PET membrane were removed using a cotton swab. The nuclei were stained with DAPI and imaged with a fluorescent microscope (Olympus slide scanner BX61VS and the vs‐asw‐s6 software) at a wavelength of 461 nm. The fold‐change of the number of cells which had migrated toward fibronectin and the negative control was calculated.

### Adhesion to fibronectin

To measure the strength of interaction between cells and fibronectin, cantilevers were coated with fibronectin. Here, we used tip‐less triangular NP‐O cantilevers with similar properties as the cantilevers mentioned above. Briefly, cantilevers were washed with EtOH, dried with N_2_, cleaned with UV/ozone for 30 min before washing them three times in PBS, incubating them for 1 h in 1 mg·mL^−1^ poly‐l‐lysine, washing them again and incubating them in 20 µg·mL^−1^ fibronectin for 1 h. Cantilevers were stored in 1× PBS at 4 °C for a maximum of 3 days and calibrated using the thermal noise method prior to use. The same AFM described above was used with an additional CellHesion module, which increases the available z‐range to 100 µm but increasing piezo response time and decreasing piezo accurateness. Cells were approached at 5 µm·s^−1^ and indented up to a maximum force of 2 nN, then the contact (between tip and sample) was held at constant height for 1 s. After, the cantilever was retracted with a velocity of 5 µm·s^−1^ for a range of 100 µm with a sample rate of 2048 Hz. Cells were treated similarly as above using the in‐house written R code, additionally defining the detachment point using similar algorithms as for the contact points. The area under the retract curve which is the work of adhesion was determined in between the retract zero‐force point and the detachment point following

(2) $$W_{\text{Adh}}=\int_{0}^{F_{\text{DP}}}F(z)dz$$

### Immunofluorescence staining

0.6 × 10^5^ primary fibroblasts were cultured on fibronectin (10 μg·mL^−1^) coated coverslips in a 12‐well dish for 24 h, before serum starvation for 3 h. Starved fibroblasts were fixed with 4% PFA for 10 min at RT, washed with PBS and permeabilised with 0.3% Triton‐X 100 for 10 min at RT. Unspecific binding sites were blocked with blocking buffer containing 2% BSA and 2% FBS for 1 h at RT. Cells were incubated with the phalloidin‐iFluor 647 reagent (Abcam, Cambridge, UK) diluted 1 : 1000 in 1% BSA in PBS for 1.5 h at RT. The cells were washed with PBS before mounting with antifade containing DAPI. DAPI and phalloidin were visualised with the Olympus slide scanner BX61VS and the vs‐asw‐s6 software. Cell size and alignment of actin filaments were analysed in a blind study by using tyche (https://tyche.expert/).

### Integrin internalisation assay

0.6 × 10^5^ cells were grown on fibronectin (10 μg·mL^−1^) coated coverslips in a 12‐well dish and serum starved for 3 h. Cells were subsequently incubated with anti‐integrin mAb 9EG7 (BD Biosciences, Franklin Lakes, NJ, USA) for 45 min at 4 °C. Cells were then processed as follows: to detect total integrin levels cells were fixed with methanol and incubated with goat anti‐rat Alexa 488‐labeled secondary antibody (Invitrogen); to detect background staining cells were acid washed (0.5 m NaCl, 0.5% acetic acid pH 2.8) twice for 6 min each, fixed and stained with goat anti‐rat Alexa 488‐labelled secondary antibody; to detect internalised integrin, cells were incubated with pre‐warmed medium at 37 °C for 60 min, acid washed, fixed and permeabilised followed by staining with goat anti‐rat Alexa 488‐labelled secondary antibody as the experimental sample. The samples were mounted using antifade containing DAPI, imaged and analysed. Mean fluorescence intensity per cell was calculated using imagej and compared between the two genotypes. Around 80–100 cells were quantified per replicate for each sample.

### Protein elasticity measurements

To measure the elasticity of Filamin A repeat 22 in both, edited and unedited versions, single FLNA^Q^ and FLNA^R^ domains were introduced into a 7‐domain titin I27 polyprotein. Titin I27 is a well‐studied immunoglobulin domain of the giant human titin protein with high mechanical stability [77, 78, 79, 80]. Human FLNA repeat 22 was cloned in pETM14 plasmid (Addgene, Watertown, MA, USA) [81] using NheI and XhoI restriction enzymes. The resulting construct flanks FLNA Ig repeat 22 with 3 titin I27 domains at the N‐terminus and 4 titin I27 domains at the C‐terminus. All proteins contained a C‐terminal cysteine which enabled covalent binding to a gold surface via thiol bonds. Glass slides were EtOH cleaned, N_2_ dried, plasma cleaned and finally covered with a thin film layer of gold (Leica, SCD). Then, 50 µL of the respective protein solutions with a concentration of around 75–100 µg·mL^−1^ were added to the slide and left to bind for 30 min. Slides were rinsed carefully with PBS. Soft triangular cantilevers (MLCT, C) with a nominal stiffness of 0.01 N·m^−1^, and resonance frequency of 7 kHz in air (around 1 kHz in liquid) were cleaned for 30 min using UV/ozone cleaning. Prior to measurements, cantilevers were calibrated using the equipartition theorem and equilibrated for 30 min near to the surface. The AFM already described was used with the only difference that the maximum piezo range was decreased to 5 µm, thus increasing accuracy and decreasing reaction time.

Measurements were performed using the force mapping/force volume mode. Depending on tip velocity, either maps with 2 µm × 2 µm and 32 × 32 pixels (1600 nm·s^−1^) or 1 × 1 and 25 × 25 pixels (400 nm·s^−1^) were performed. The sample surface was approached with the respective speed and indented to a force of 1 nN, the residence time of the tip was set to 2 s at constant height before it was retracted. The curve length was set to 800 nm with a sampling rate according to the velocity of the measurement. For each protein (FLNA^R^, FLNA^Q^) and each speed (400 and 1600 nm·s^−1^) at least 6000 measurements were performed in such a way. In addition, control measurements were performed at both extension rates using a construct containing only the I27 domain of titin. Measurements were sorted according to the guidelines defined in literature [77]. Briefly, a sawtooth like pattern of mechanical protein unfolding was expected, with the unfolding of domains bearing low mechanical strengths happening before those of higher mechanical strength (according to probabilistic unfolding theory like Bell–Evans) [39, 40]. Such curves were fitted using the worm‐like chain entropic polymer unfolding model (using the jpkdp) following the relation

(3) $$F\left(x\right)=\frac{k_{b}T}{p}\left[\frac{1}{4}{\left(1‐\frac{x}{L_{c}}\right)}^{‐2}‐\frac{1}{4}+\frac{x}{L_{c}}\right]$$

connecting the unfolding force of a segment in a polymer with the respective contour length *L*_c_. All fittings were performed with a defined persistence length *p* of 0.4 nm, according to the average length of amino acid stretching. I27 domain of titin was used as an internal reference protein in the measurements, with a defined contour length of around 27 nm and an unfolding force of 215 pN at 400 nm·s^−1^ and 250 pN at 1600 nm·s^−1^.

### Flow cytometry

Primary fibroblasts were grown on 0.1% gelatine‐coated 10 cm dishes until confluence. Then, cells were trypsinised, transferred to fibronectin (10 µg·mL^−1^) coated 10 cm dishes and cultured for 24 h. Following activation, fibroblasts were starved for 3 h before trypsinisation and fixing with 4% PFA for 10 min at RT. Before antibody staining, cells were permeabilised with 0.3% Triton X‐100 for 10 min at RT. Cells were incubated with the primary antibodies rabbit anti‐integrin β1 polyclonal antibody (Cell Signalling, Danvers, MA, USA), rat anti‐integrin β1 mAb 9EG7 (BD Biosciences) diluted 1 : 100 in 1% BSA in PBS for 1 h at RT. After washing with PBS cells were incubated with the corresponding secondary antibodies, goat anti‐rat Alexa 488‐ and goat anti‐rabbit Alexa 647‐labelled antibodies (Invitrogen) diluted 1 : 300 in 1% BSA in PBS in the dark for 30 min at RT. Cells were washed with PBS before FACS analysis, using the CytoFLEX S flow cytometer (Beckman Coulter, Krefeld, Germany).

### Western blotting

Both fibroblasts and M2 cells were harvested and lysed with 2× SDS buffer. The samples were then sonicated on ice and centrifuged at 17 000 g. for 20 min at 4 °C. The supernatant was boiled and loaded on SDS/PAGE gels. For fibroblast samples, the proteins were detected either with homemade rabbit anti‐FLNA polyclonal antibody and anti‐GAPDH antibody that served as a loading control. For M2 cells, the membranes were incubated with anti‐myc mAb 9E10 [82] and anti‐GAPDH that served as a loading control. All blots were detected by chemiluminescence and images using a CCD camera on a Fusion‐FX (Fisher Biotec, West Perth, WA, Australia). Gel densitometric analyses were done using imagej. FLNA expression (fibroblasts) and myc expression (M2) was compared between the unedited and edited samples after normalising to GAPDH expression.

### RT‐PCR and determination of FLNA editing levels

Total RNA was isolated from cells with PeqGOLD TRIzol reagent (PEQLAB Biotechnologie GmbH, Erlangen, Germany) using the manufacturer's protocol. After DNAseI treatment, cDNAs were synthesised using M‐MLV Reverse Transcriptase kit (Invitrogen) and random hexamer primers. A FLNA cDNA fragment spanning spliced exons 42–43 was amplified from synthesised cDNAs, gel eluted and sequenced to check editing levels. Primers used for amplification were; forward primer (5′ GTCAAGTTCAACGAGGAGCAC 3′) and reverse primer (5′ GTGCACCTTGGCATCAATTGC 3′).

### Strep tag purification

One litre bacterial cultures were grown till an OD_660_ of 0.6 and then induced with 1 mm of IPTG and grown for another 3 h. Thereafter, the cells were harvested, sonicated and checked for presence of protein in supernatant and pellet fraction. The supernatant was loaded onto a Strep‐tag column and was then washed with 4 column volumes of wash buffer (100 mm tris HCl pH 8, 150 mm NaCl, 1 mm EDTA). To elute the protein, 6× 0.5 column volumes of Buffer E (100 mm tris HCl pH 8, 150 mm NaCl, 1 mm EDTA, 2.5 mm desthiobiotin) were used. Part of the fractions were checked on SDS/PAGE using Coomassie staining. Since the gel showed multiple bands, a size exclusion chromatography was done to remove contaminating low molecular weight bands.

### Size exclusion chromatography

For size exclusion chromatography, the fractions that contained the protein of interest were pooled and concentrated using Amicon® ultra 2 mL centrifugal filters (Merck Millipore, Burlington, MA, USA) with a 10 kDa cut‐off. For equilibration, the column (Superdex 200 10/300 GL from GE Healthcare, Chalfont, UK) was flushed with 30 mL 1× PBS with a flowrate of 0.5 mL·min^−1^. The sample was injected and fractions were collected. Fractions containing the protein of interest (2 mL) were further concentrated to 200 µL using Amicon® ultra 2 mL centrifugal filters and quantified by densitometric analyses using known amounts of purified BSA. After estimating the concentrations of both FLNA^Q^ and FLNA^R^ by gel densitometry, the samples were subjected to AFM studies.

### Statistical analysis

The cell migration, spreading data, actin organisation and integrin internalisation data were analysed using student’s *t*‐test. *P* < 0.05 was considered to be statistically significant. For AFM‐based experiments, all datasets calculated as described above were first checked for outliers and normality. Then, statistical properties (mean, median, SD and SE) were calculated. For datasets with normal distribution, mean plus/minus standard error are given, for non‐normal‐distributed data fitting was performed. We found that log‐normal distributions fitted best to most of our datasets (R2 above 0.9 of the histograms). In addition, we performed log‐normal fittings to describe statistical properties of data. For non‐normal distributed datasets we performed non‐parametric Mann–Whitney tests to determine significance. Significance is given as alpha = 0.05 (*P < 0.05; **P < 0.01; ***P < 0.001).

## Conflict of interest

The authors declare no conflict of interest.

## Author contributions

MJ, AW, KM, GM, JD, OT, MS performed experiments. MJ, AW, KM, JD, JLT‐H and MFJ planned experiments. MJ, AW, KM, GM, JD, AB, JLT‐H and MFJ evaluated data. MJ, KM and AW performed statistical analysis. MJ, AW, KM and MFJ wrote the manuscript.

### Peer review

The peer review history for this article is available at https://publons.com/publon/10.1111/febs.16391.

## Supporting information


**Data S1.** Raw data actin aligment.

**Data S2.** Raw data cell spreading.

**Data S3.** Raw data to integrin β1 expression and internalization in primary fibroblasts (figure 7) and stable M2 cells (figure s7).

## Data Availability

No online data are deposited for this manuscript.
